# Effects of an *Astragalus membranaceus* Stem and Leaf-*Angelica sinensis* Stem and Leaf Mixture on Serum Parameters, Gut Microbiota, and Metabolomic Profiles in Simmental Weaned Bull Calves

**DOI:** 10.3390/vetsci13050414

**Published:** 2026-04-23

**Authors:** Hongya Li, Nianshou Zhao, Min Yang, Yongli Hua, Yanming Wei, Peng Ji

**Affiliations:** 1 College of Veterinary Medicine, Gansu Agricultural University, Lanzhou 730070, China; lhyning1116@163.com (H.L.); zhaonianshou@126.com (N.Z.); weiym@gsau.edu.cn (Y.W.); 2 College of Science, Gansu Agricultural University, Lanzhou 730070, China

**Keywords:** *Astragalus membranaceus* stem and leaf–*Angelica sinensis* stem and leaf mixture, calves, gut microbiota, short-chain fatty acids, serum metabolome

## Abstract

This study evaluated the effects of an *Astragalus membranaceus* stem and leaf–*Angelica sinensis* stem and leaf mixture (AASL) as a medicinal feed supplement on immune function, antioxidant status, inflammatory responses, gut microbiota and the serum metabolome in weaned Simmental bull calves. Calves were fed diets containing different levels of AASL, and serum immunoglobulins, inflammatory cytokines, and antioxidant indices were determined. In addition, fecal short-chain fatty acid (SCFA) concentrations, gut microbiota composition, and serum metabolic profiles were analyzed, followed by correlation analyses among the microbiota, SCFAs and metabolites. The results showed that AASL was rich in crude protein, crude fat and trace elements. 4% AASL supplementation increased serum immunoglobulin (IgG and IgM) levels, decreased tumor necrosis factor-alpha (TNF-α), interleukin-6 (IL-6) and interleukin-1 beta (IL-1β) levels, and enhanced superoxide dismutase (SOD), glutathione (GSH) and total antioxidant capacity (T-AOC) activities (*p* < 0.01 or *p* < 0.001), indicating improved immune and antioxidant status and attenuated inflammatory responses. AASL also enriched beneficial bacterial genera, suppressed potentially harmful taxa, and increased SCFA concentrations. Differential metabolites were mainly enriched in tryptophan metabolism, lipid metabolism, neuroactive ligand-receptor interaction, sphingolipid signaling, and ATP-binding cassette (ABC) transporter pathways. Integrated microbiota metabolite analysis further suggested that AASL improved host metabolic status through the coordinated regulation of gut microbiota, SCFAs production and related metabolic pathways. Overall, AASL shows promise as a functional feed supplement for improving calf health.

## Simple Summary

Weaned calves are highly susceptible to diarrhea, oxidative stress, and immune dysfunction during early growth, which can impair development and increase economic losses. Plant-derived feed additives are considered promising alternatives to antibiotics, as they can improve gut health and immune function. In this study, we evaluated the effects of a compound mixture of *Astragalus membranaceus* stem and leaf–*Angelica sinensis* stem and leaf mixture (AASL) in weaned calves, focusing on immune and inflammatory status, antioxidant capacity, gut microbiota, short-chain fatty acids (SCFAs), and serum metabolites. The results indicate that AASL may enhance calf health by modulating the gut microbiota and microbial metabolites, as well as improving host metabolic pathways, providing a scientific basis for its application as a functional feed additive during the calf-rearing period.

## 1. Introduction

The calf-rearing period is a critical stage for growth and development, immune establishment, and maturation of gastrointestinal function in ruminants. Owing to multiple stressors, including weaning, dietary transition, environmental changes, and pathogen exposure, calves are highly susceptible to diarrhea, growth retardation, and immune dysfunction, which in turn lead to increased morbidity and economic losses. Neonatal calf diarrhea and bovine respiratory disease remain major causes of morbidity and mortality in calves worldwide, and diarrheic calves are often accompanied by marked disturbances in the gut microbiota [1]. In addition, studies have shown that diarrhea during the calf-rearing period can induce persistent alterations in the fecal microbiota of weaned calves, further indicating that gut microbial dysbiosis is closely associated with calf health status [2]. In recent years, the rapid development of multi-omics technologies has provided new approaches for elucidating the mechanisms of feed additives [3,4]. Among these, 16S rRNA sequencing enables comprehensive characterization of changes in gut microbial structure, whereas untargeted metabolomics systematically captures disturbances in host metabolic profiles. The integrated analysis of these two approaches can therefore help clarify the potential mechanisms of feed additives from a systems biology perspective [3,5].

Against the background of antibiotic reduction, restriction, and green livestock production, phytogenic feed additives have attracted considerable attention as potential alternatives to antibiotics because they contain bioactive constituents such as polysaccharides, flavonoids, and phenolic acids [6,7]. Studies have shown that these additives may promote animal health and production performance in pigs, poultry, and some ruminants by modulating gut microbiota composition, alleviating oxidative stress, and regulating inflammatory and immune responses [8]. However, the biological effects of phytogenic additives are influenced by multiple factors, including plant source, harvest stage, supplementation level, processing method, and animal species; therefore, their efficacy should be evaluated in specific contexts. Phytogenic additives have also been reported to improve diarrhea-related symptoms, gut health, and immune status in calves to a certain extent [9], indicating promising potential for their application in the health management of young ruminants. Nevertheless, studies on their efficacy in weaned calves remain relatively limited.

*Astragalus membranaceus* and *Angelica sinensis* are well-known medicinal and edible plants rich in polysaccharides, flavonoids, phenolic acids, volatile oils, and other plant secondary metabolites, exhibiting antioxidant, anti-inflammatory and immunomodulatory activities. Among them, the antioxidant effects of *Astragalus membranaceus* have been systematically reviewed, with the bioactive basis primarily attributed to flavonoids, polysaccharides and saponins [10]. In ruminants, *Astragalus membranaceus* has been reported as a feed additive to improve serum biochemical parameters, immune function, and production-related performance in dairy cows [11]. Meanwhile, supplementation with the aerial parts of *Angelica sinensis* has been shown to enhance growth performance, antioxidant capacity, and intestinal health in poultry, suggesting that its non-conventional medicinal parts also possess considerable potential for feed development [12]. In addition to plant-derived bioactive compounds, short-chain fatty acids (SCFAs) serve as crucial mediators linking dietary interventions, gut microbiota dynamics, and host health outcomes. Emerging reviews have highlighted that SCFAs are not only major fermentation products of gut microbiota but also play pivotal roles in maintaining intestinal barrier function, regulating mucosal immunity, and mediating host metabolic responses, particularly during the intestinal immune maturation process in young ruminants [13]. Therefore, integrating the gut microbiota, SCFAs, and host metabolic profiling offers a more systematic approach to elucidating the underlying biological mechanisms of phytogenic feed additives.

Based on the core concept of “dual tonification of Qi and blood” in traditional veterinary medicine, *Astragalus membranaceus* stem and leaf (AMSL) is considered a representative Qi-tonifying herb and is believed to elevate Yang and strengthen body defenses. *Angelica sinensis* stem and leaf (ASSL) is regarded as an important blood-tonifying herb and is traditionally used to promote blood circulation and remove blood stasis.When combined, Qi supports blood, and blood carries Qi, forming a classic synergistic interaction between “Qi herbs” and “blood herbs”. Previous studies have largely focused on the individual application of *Astragalus membranaceus* stem and leaf or *Angelica sinensis* stem and leaf, with relatively few systematic investigations in calves. In addition, there is a lack of integrated analyses encompassing gut microbiota, SCFAs, and serum metabolomic profiles, and studies combining microbiome and metabolome data to explore potential mechanisms remain scarce. To address this gap, the present study formulated an *Astragalus membranaceus* stem and leaf–*Angelica sinensis* stem and leaf mixture (AASL) as a feed additive for weaned calves. We hypothesized that dietary supplementation with AASL may improve antioxidant capacity, alleviate inflammation, and immune function in weaned calves by modulating the gut microbiota and serum metabolic profiles, thereby contributing to health maintenance in weaned calves. We systematically evaluated its effects on body weight, blood routine parameters, serum inflammatory cytokines, immune factors, antioxidant indices, SCFAs, gut microbiota composition and untargeted metabolomic profiles. Furthermore, integrative correlation analysis between microbiome and metabolome was performed to explore the underlying mechanisms. This study aims to provide a theoretical basis for the resource utilization of herbal by-products and contribute to the green and healthy breeding of calves.

## 2. Materials and Methods

### 2.1. Animals and Experimental Design

This experiment was conducted at Wanhe Grassland Animal Husbandry Technology Development Co., Ltd. in Zhangye City, China. The stem and leaf materials of *Astragalus membranaceus* and *Angelica sinensis* were collected from Min County, air-dried and subsequently pulverized. The pulverized materials were uniformly mixed at a ratio of 5:1 (*Astragalus membranaceus* stem and leaf: *Angelica sinensis* stem and leaf) to obtain the AASL. A total of 80 Simmental weaned bull calves, 5 months of age and with similar initial body weight (approximately 135 ± 5 kg), were used in this experiment. Before weaning, the calves were housed in individual pens, and after weaning they were group-housed for 2 months. At the beginning of the trial, 80 calves with comparable body weight were selected and randomly allocated to four treatment groups, with 20 calves in each group. Each group was maintained in a separate pen. Based on our research group’s preliminary exploratory studies in mice, and taking into account the practical feasibility, palatability, and the need for a dose gradient in weaned calves, three supplementation levels—low, medium, and high—were ultimately selected. The grouping and corresponding AASL doses are as follows: basal diet group (CON group), basal diet supplemented with 2% AASL group, basal diet supplemented with 4% AASL group, and basal diet supplemented with 8% AASL group. The experimental diets were fed consecutively for 7 days. To maintain approximate equivalence in overall energy and nitrogen content across all groups, the corresponding amount of AASL was used to proportionally replace part of the basal fiber sources, including corn silage and wheat straw. The nutritional composition of the basal diet and AASL is shown in Table 1, and the ingredient composition of the experimental diets is shown in Table 2.

On day 8, prior to morning feeding, blood and fecal samples were collected. On day 15, before morning feeding, fasting body weight was measured again. Calves were fed a precision total mixed ration with ad libitum access to feed, but individual feed intake was not separately determined to avoid additional handling stress during the post-weaning period. All animal experimental procedures were strictly conducted in accordance with internationally recognized animal welfare and ethical guidelines and were approved by the Animal Ethics Review Committee of Gansu Agricultural University (Approval No.: GSAU-Eth-VMC-2025-054). The experiment was designed with a 7-day feeding period to evaluate the short-term regulatory effects of AASL on antioxidant status, inflammatory responses, immune function, and gut microbiota-related parameters.

### 2.2. Determination of Major Bioactive Fractions in AASL

*Astragalus membranaceus* stems and leaves and *Angelica sinensis* stems and leaves were collected, air-dried indoors, ground, and passed through a 60-mesh sieve. The two materials were then thoroughly mixed at a ratio of 5:1 (*w*/*w*). The mixture was soaked in eightfold volume of water for 30 min and extracted twice, with each extraction lasting 60 min. After filtration, the combined filtrates were centrifuged at 3000 rpm, concentrated under reduced pressure, and freeze-dried to obtain the AASL extract.

The contents of total sugars, total flavonoids, and total saponins in AASL were determined using microplate reader-based colorimetric assays. Total sugars were quantified by the phenol-sulfuric acid method using glucose as the reference standard. Total flavonoids were determined by the NaNO_2_-Al (NO_3_)_3_-NaOH colorimetric method using rutin as the reference standard. Total saponins were quantified by the vanillin-perchloric acid method using ginsenoside Rb1 as the reference standard. Calibration curves were established with the corresponding standards, and the contents of these bioactive fractions were calculated accordingly. All measurements were performed in triplicate, and the results are expressed as mean ± standard deviation (SD).

### 2.3. Growth Performance and Hematological Analysis

Calves were weighed before the morning feeding on day 1 of the experimental period after an overnight fast to obtain the initial body weight. Following 7 days of AASL supplementation, all groups were fed the basal diet alone for an additional 7 days. On day 15, before the morning feeding and after fasting, the calves were weighed again to obtain the final body weight. Average daily gain (ADG) was calculated using the following equation:

ADG (kg/d) = (final body weight − initial body weight)/14.

For hematological analysis, jugular venous blood samples were collected from each calf before the morning feeding on day 8. Blood samples were transferred into EDTA-containing anticoagulant tubes and used for the determination of peripheral blood parameters, including white blood cell count (WBC), granulocyte count (Gran), lymphocyte count (LYM), and monocyte count (MON). Leukocyte differential parameters were determined using an automated hematology analyzer (BC-30 Vet, Mindray Animal Medical Technology Co., Ltd., Shenzhen, China).

### 2.4. Determination of Serum Anti-Inflammatory, Antioxidant and Immune Parameters

Additional blood samples were collected into 5 mL non-anticoagulant tubes. After standing at room temperature for 2 h, the samples were centrifuged at 3500 rpm for 20 min, and the serum supernatant was collected and stored at −80 °C until further analysis. The serum samples were used for the determination of anti-inflammatory, antioxidant and immune-related parameters. Commercial assay kits for tumor necrosis factor-α (TNF-α; Cat. #RX1600738B), interleukin-6 (IL-6; Cat. #RX1600853B), interleukin-1β (IL-1β; Cat. #RX1600856B), immunoglobulin G (IgG; Cat. #RX1600039B), immunoglobulin M (IgM; Cat. #RX1600803B), and immunoglobulin A (IgA; Cat. #RX1600805B) were purchased from Ruixin Biotechnology Co., Ltd. (Quanzhou, China). Commercial kits for glutathione (GSH; Cat. #A006-2-1), catalase (CAT; Cat. #A007-1-1), superoxide dismutase (SOD; Cat. #A001-3-2), and malondialdehyde (MDA; Cat. #A003-1) were obtained from Nanjing Jiancheng Bioengineering Institute (Nanjing, China). All assays were performed according to the manufacturers’ instructions.

### 2.5. Gut Microbial Diversity Analysis

Fresh fecal samples (10 g) were collected directly from the rectum of each calf before the morning feeding on day 8 of the trial. Each sample was placed into a sterile cryotube, labeled with the sampling time and sample identification number, immediately snap-frozen in liquid nitrogen, and then stored at −80 °C until further analysis. These samples were used for both fecal microbiota diversity analysis and SCFAs determination.

A total of 20 fecal samples were submitted to Majorbio Bio-Pharm Technology Co., Ltd. (Shanghai, China) for total genomic DNA extraction. The V3–V4 hypervariable region of the 16S rRNA gene was amplified by PCR using the barcode-tagged forward primer 338F (5′-ACTCCTACGGGAGGCAGCAG-3′) and reverse primer 806R (5′-GGACTACHVGGGTWTCTAAT-3′). Purified PCR products were then used for library construction with the NEXTFLEX Rapid DNA-Seq Kit. Raw sequencing reads were assigned to individual samples according to barcode sequences, followed by quality filtering and orientation correction. High-quality sequences were clustered into operational taxonomic units (OTUs) at 97% sequence similarity using UPARSE (version 7.1), and chimeric sequences were removed during the clustering process. To minimize biases caused by sequencing depth, all samples were rarefied to 47,464 sequences per sample.

Alpha diversity indices and beta diversity analyses were calculated using Mothur software (version 1.30.2), and visualization was performed in R (version 3.3.1). Principal coordinate analysis (PCoA) based on Bray–Curtis distances, combined with ANOSIM and Adonis tests, was used to evaluate overall differences in bacterial community structure among samples. In addition, community bar plots and heatmaps were generated to illustrate the distribution patterns of dominant taxa.

### 2.6. Determination of SCFAs

SCFAs were quantified by gas chromatography (GC). The GC system consisted of an Agilent 8890 GC (Agilent Technologies, Santa Clara, CA, USA) equipped with a DB-FFAP capillary column, with high-purity nitrogen (>99%) used as the carrier gas at a flow rate of 1.0 mL/min. The injector temperature was set at 280 °C, and the flame ionization detector temperature was set at 300 °C. The oven temperature program was as follows: an initial temperature of 60 °C was maintained for 2 min; the temperature was then increased from 60 to 140 °C at a rate of 10 °C/min, followed by a further increase to 170 °C at 3 °C/min. Samples were injected manually in split mode with a split ratio of 20:1, and the injection volume was 1 μL. Mixed SCFA standard solutions containing acetic acid, propionic acid, isobutyric acid, n-butyric acid, n-valeric acid were prepared at a ratio of 20:5:2.5:5:0.5 to establish standard curves and corresponding linear regression equations. Acetic acid (Lot #A801299, CAS 64-19-7), propionic acid (Lot #C11714091, CAS 79-09-4), isobutyric acid (Lot #C12177949, CAS 79-31-2), n-butyric acid (Lot #C11882450, CAS 107-92-6), and n-valeric acid (Lot #C11473010, CAS 503-74-2) were purchased from Macklin Biochemical Co., Ltd. (Shanghai, China). For sample preparation, 0.4 g of fecal sample was weighed into a 2 mL centrifuge tube and mixed with two volumes of deionized water, followed by vortexing for 1 min. After standing for 20 min, the sample was centrifuged at 15,000 rpm for 10 min at 4 °C. Subsequently, 247.5 μL of the supernatant was transferred to a new tube and mixed with 2.5 μL of n-butanol dilution solution, followed by vortexing. The mixture was then filtered through a 0.22 μm membrane filter, and 1 μL of the filtrate was injected into the GC system for analysis. Target compounds in the fecal samples were identified according to their retention times relative to those of the corresponding standards. SCFAs concentrations in the sample extracts were calculated using the linear regression equations derived from the standard curves.

### 2.7. Untargeted Metabolomics Analysis

Based on the preliminary results, the optimal-dose group and the CON group were selected for untargeted metabolomics analysis. A total of 20 serum samples were submitted to Majorbio Bio-Pharm Technology Co., Ltd. (Shanghai, China) for LC–MS analysis, which was performed on a UHPLC-Q Exactive system (Thermo Fisher Scientific, Waltham, MA, USA). Quality control (QC) samples were prepared by mixing equal volumes of different biological samples. During LC-MS analysis, one QC sample was inserted after every 5–10 test samples to monitor the repeatability of the entire analytical workflow and the stability of the instrument. After data acquisition, the raw LC-MS data were imported into the metabolomics processing software Progenesis QI for baseline filtering, peak detection, integration, retention time correction, and peak alignment, generating a data matrix containing retention time, mass-to-charge ratio (m/z), and peak intensity information. MS peak intensities were normalized using the total sum normalization method. Variables with a relative standard deviation (RSD) greater than 30% in QC samples were subsequently removed, and the remaining data were log10-transformed to obtain the final data matrix for downstream statistical analysis.

The preprocessed data matrix was analyzed using principal component analysis (PCA) and partial least squares discriminant analysis (PLS-DA) implemented in the R package ropls (Version 1.6.2). Model stability was assessed using seven-fold cross-validation. Differential metabolites were identified based on the variable importance in projection (VIP) values from the PLS-DA model and Student’s *t*-test, using thresholds of VIP > 1 and *p* < 0.05. Python Identified differential metabolites were annotated against the KEGG database, and pathway enrichment analysis was performed using the scipy.stats module in Python, based on SciPy (version 1.0.0).

### 2.8. Statistical Analysis

Peripheral hematological parameters, serum inflammatory, immune, and antioxidant indices, as well as fecal SCFA data, were analyzed using IBM SPSS Statistics 26.0 (IBM Corp., Armonk, NY, USA). Results are presented as mean ± SD. Statistical significance among groups was assessed by one-way analysis of variance, followed by Tukey’s multiple comparison test. A value of *p* < 0.05 was considered statistically significant, while *p* < 0.01 or *p* < 0.001 was considered highly significant.The data plots were generated using GraphPad Prism 9.5.0 (GraphPad Software, Boston, MA, USA).

For gut microbiota analysis, intergroup differences in alpha diversity were assessed using the Wilcoxon rank-sum test. Differential taxa were identified by LEfSe (Linear Discriminant Analysis Effect Size, version 1.0; Huttenhower Lab, Boston, MA, USA) analysis with thresholds of LDA > 2 and *p* < 0.05. For differential metabolites in serum metabolomics and multiple comparisons involving gut microbiota, *p*-values were adjusted using the Benjamini–Hochberg method to control the false discovery rate (FDR), with *p* < 0.05 considered statistically significant after correction. Correlation analyses were performed using the Spearman rank correlation method.

## 3. Results

### 3.1. Contents of Major Bioactive Fractions in AASL

**Figure 1:**
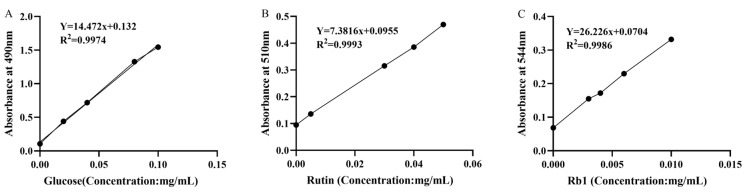
Standard curves for the determination of total sugars, total flavonoids, and total saponins in AASL. (**A**) Glucose standard curve for total sugars. (**B**) Rutin standard curve for total flavonoids. (**C**) Ginsenoside Rb1 standard curve for total saponins.

The major bioactive fractions in the AASL sample used in this study were quantitatively determined, and the results are shown in Table 3. The contents of total sugars, total flavonoids, and total saponins were 2.24 ± 0.09%, 0.12 ± 0.01%, and 1.22 ± 0.40%, respectively, based on the original powder. The standard curves for glucose, rutin, and ginsenoside Rb1 are presented in Figure 1. All three calibration curves exhibited good linearity, demonstrating that the methods were reliable for quantifying the corresponding bioactive fractions in AASL.

### 3.2. Effects of Dietary AASL Supplementation on Growth Performance in Calves

The initial body weight, final body weight, and average daily gain of calves in each group are shown in Table 4. Compared with the control group, supplementation with 4% AASL significantly increased the average daily gain of calves (*p* < 0.05), whereas no significant differences were observed in the other supplementation groups (*p* > 0.05).

### 3.3. Effects of AASL Supplementation on Peripheral Hematological Parameters in Calves

**Figure 2:**
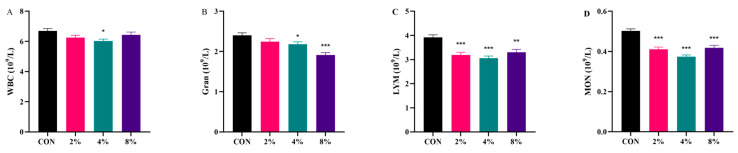
Effects of dietary supplementation with AASL on peripheral hematological parameters in calves. (**A**) White blood cell (WBC); (**B**) Granulocyte (Gran); (**C**) Lymphocyte (LYM); (**D**) Monocyte (MON). Compared with the CON group, * indicates significance at *p* < 0.05, ** indicates significance at *p* < 0.01, and *** indicates significance at *p* < 0.001.

According to the hematological results (Figure 2), compared with the CON group, supplementation with 4% AASL significantly reduced WBC and Gran levels (*p* < 0.05), while supplementation with 8% AASL significantly decreased the Gran level (*p* < 0.01). In addition, all AASL-treated groups showed reduced LYM and MON levels compared with the CON group, with highly significant differences (*p* < 0.01 or *p* < 0.001). It suggests that supplementation with 4% AASL may be associated with a reduced inflammatory burden or an alleviated inflammatory status. The detailed numerical values corresponding to Figure 2 are provided in Supplementary Table S1.

### 3.4. Effects of Dietary AASL Supplementation on Serum Immune and Inflammatory Parameters in Calves

**Figure 3:**
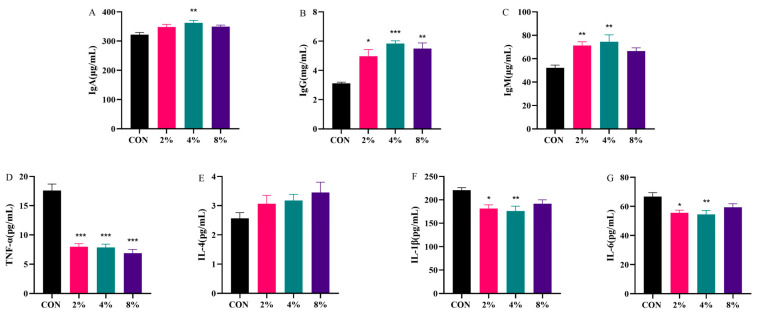
Effects of different doses of treatment on serum immunoglobulin and cytokine levels in calves. (**A**) Immunoglobulin A (IgA); (**B**) Immunoglobulin G (IgG); (**C**) Immunoglobulin M (IgM); (**D**) Tumor necrosis factor-α(TNF-α); (**E**) Interleukin-4 (IL-4); (**F**) Interleukin-1β (IL-1β); (**G**) Interleukin-6 (IL-6). * Indicates significance at *p* < 0.05, ** indicates significance at *p* < 0.01, and *** indicates significance at *p* < 0.001.

As shown in Figure 3, compared with the CON group, supplementation with 4% AASL significantly increased the serum levels of IgA, IgM and IgG in calves (*p* < 0.01 or *p* < 0.001), with the most pronounced effect observed for IgG (*p* < 0.01). Supplementation with 2% AASL also significantly increased the serum levels of IgM and IgG (*p* < 0.05 or *p* < 0.01). In contrast, no significant differences were observed between the other supplementation groups and the CON group (*p* > 0.05).

Compared with the CON group, supplementation with 2% and 4% AASL significantly reduced the levels of IL-6 and IL-1β (*p* < 0.05 or *p* < 0.01), while all AASL-supplemented groups significantly decreased TNF-α levels (*p* < 0.01 or *p* < 0.001). AASL supplementation also increased IL-4 levels, although the change was not statistically significant (*p* > 0.05). These results suggest that calves in the CON group may still be susceptible to stress under normal feeding conditions, resulting in a low-grade inflammatory response. AASL intervention altered the levels of IgG, IgM, and certain inflammatory cytokines, indicating that it may exert a modulatory effect on immune-related responses in calves. The detailed numerical values corresponding to Figure 3 are provided in Supplementary Table S2.

### 3.5. Effects of Dietary AASL Supplementation on Serum Antioxidant Parameters in Calves

**Figure 4:**
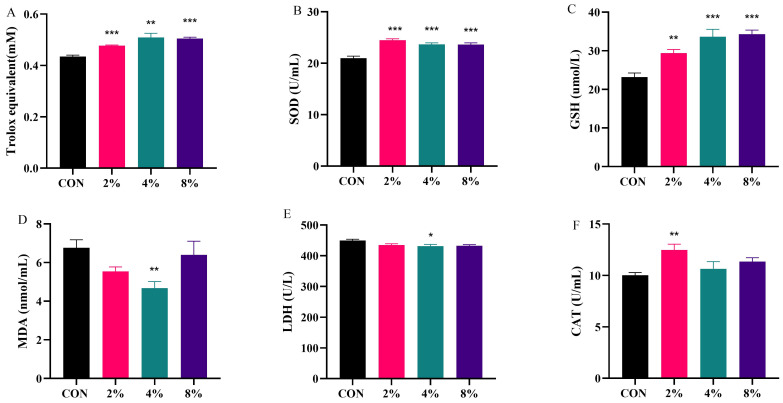
Effects of dietary AASL supplementation on serum antioxidant parameters in calves. Note: (**A**) Total antioxidant capacity(T-AOC); (**B**) Superoxide dismutase (SOD); (**C**) Glutathione (GSH); (**D**) Malondialdehyde (MDA); (**E**) Lactate dehydrogenase (LDH); (**F**) Catalase (CAT). * Indicates significance at *p* < 0.05, ** indicates significance at *p* < 0.01, and *** indicates significance at *p* < 0.001.

As shown in Figure 4, compared with the CON group, supplementation with 4% AASL significantly reduced the levels of the oxidative stress markers MDA and LDH (*p* < 0.05 or *p* < 0.01). In addition, all AASL-treated groups significantly increased T-AOC, as well as SOD and GSH activities(*p* < 0.01 or *p* < 0.001), whereas supplementation with 2% AASL significantly increased CAT activity (*p* < 0.01). These results indicate that AASL supplementation can, to some extent, alleviate stress responses caused by environmental and other factors in calves and help maintain overall physiological health. The detailed numerical values corresponding to Figure 4 are provided in Supplementary Table S3.

### 3.6. Effects of AASL Supplementation on Gut Microbiota in Calves

#### 3.6.1. OTU Clustering Analysis

**Figure 5:**
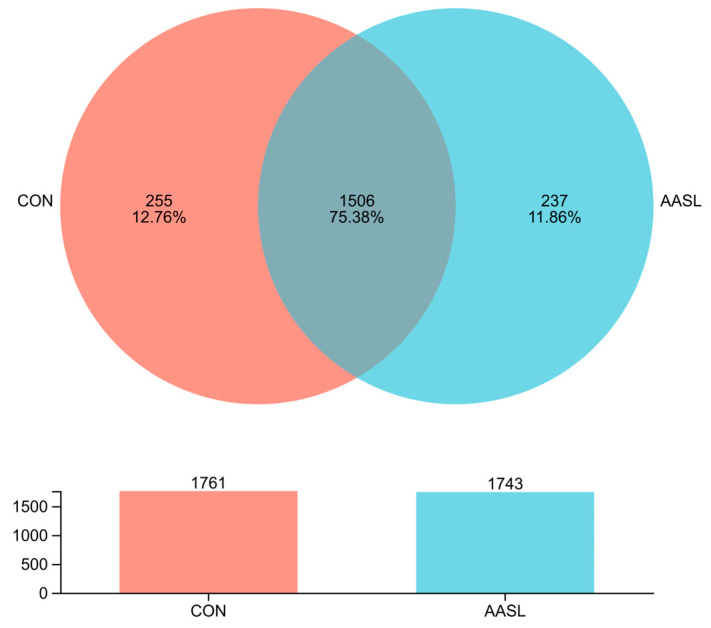
Venn diagram showing the number of common and unique OTUs of gut microbiota in calves fed with AASL supplementation.

The OTU analysis results (Figure 5) showed that the CON and AASL groups shared 1506 microbial taxa, accounting for 75.38% of the total observed taxa. In addition, AASL-specific taxa accounted for 13.59%, whereas CON-specific taxa accounted for 14.48%. These findings indicate that dietary AASL supplementation did not disrupt the original core gut microbiota, suggesting good safety and compatibility with the intestinal microecological environment.

#### 3.6.2. Alpha Diversity Analysis

**Figure 6:**
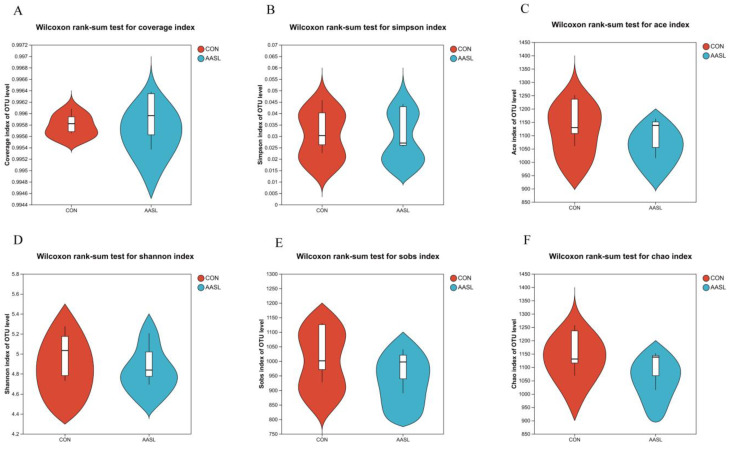
Effects of dietary AASL supplementation on the alpha diversity of the gut microbiota in calves: (**A**) Coverage index; (**B**) Simpson index; (**C**) Ace index; (**D**) Shannon index; (**E**) Sobs index; and (**F**) Chao index.

The alpha diversity analysis showed that, compared with the CON group, the AASL group exhibited an increased Coverage index, indicating improved sequencing coverage and more complete microbial community information, thereby enhancing the reliability of the sequencing results. In contrast, the Ace, Chao, and Sobs indices were lower in the AASL group than in the CON group, suggesting reduced microbial richness and diversity. However, the Simpson and Shannon indices were generally similar between the two groups, indicating comparable proportions and distribution patterns of dominant taxa (Figure 6). These results suggest that AASL intervention did not markedly alter the overall species diversity composition of the intestinal microbiota in calves.

#### 3.6.3. Beta Diversity Analysis

**Figure 7:**
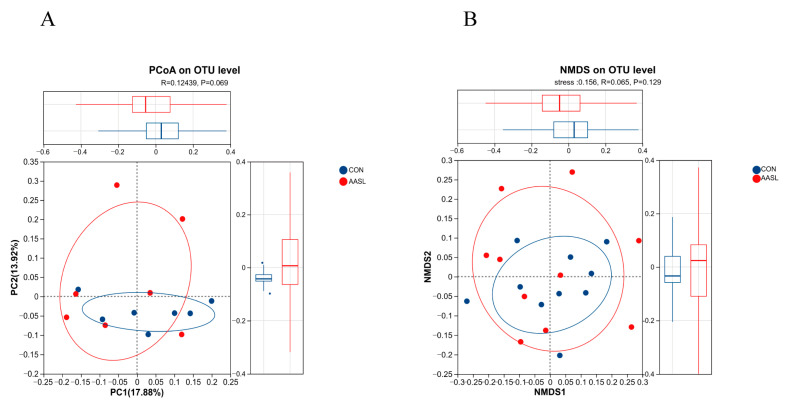
Effect of AASL supplementation on the beta diversity of gut microbiota in calves. (**A**) Principal coordinate analysis (PCoA); (**B**) Non-metric Multidimensional Scaling (NMDS) analysis. Red represents the CON group, and blue represents the AASL group. Each dot represents an individual sample, and each ellipse indicates the distribution range of samples within a group.

As shown in Figure 7, the first two principal coordinates in the PCoA explained 17.88% and 13.92% of the total community variation, respectively. The two groups exhibited a certain degree of separation in the ordination space; however, the difference between groups did not reach statistical significance (R = 0.12439, *p* = 0.069) (Figure 7A). The NMDS results were generally consistent with those of the PCoA. Although the two groups also showed a tendency toward separation in overall community structure, substantial overlap was still observed, and the difference remained non-significant (R = 0.065, *p* = 0.129; stress = 0.156) (Figure 7B). It suggests that AASL may influence the overall structure of the gut microbiota.

#### 3.6.4. Differential Gut Microbiota

**Figure 8:**
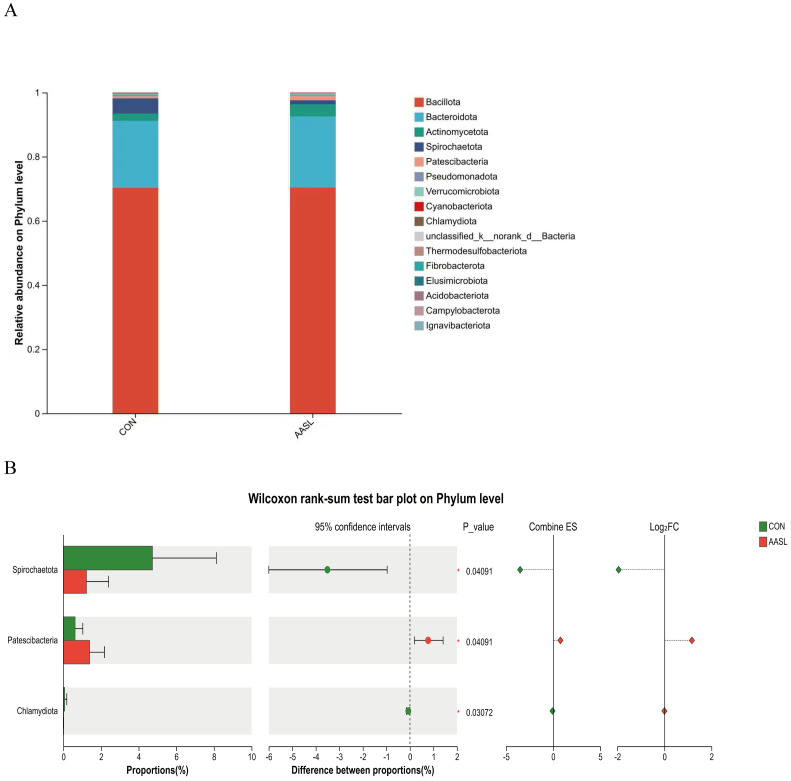
Effects of dietary supplementation with AASL on the phylum-level microbial community structure in calves: (**A**) Phylum-level microbial community composition; Different colors represent different phylum. (**B**) Differential phylum of the microbial community at the phylum level. Green represents the CON group and red represents the AASL group. Dots indicate the estimated differences, horizontal lines indicate the 95% confidence intervals, diamonds indicate the effect size and Log_2_FC, and the vertical dashed line indicates the zero reference line. * Indicates a significant difference (*p* < 0.05).

At the phylum level, the gut microbiota of both groups was predominantly composed of Bacillota, Bacteroidota and Actinomycetota, which represented the dominant bacterial phylum in the intestinal community (Figure 8A). Compared with the CON group, the relative abundances of Spirochaetota and Chlamydiota were significantly decreased in the AASL group, whereas Patescibacteria was significantly increased (Figure 8B).

**Figure 9:**
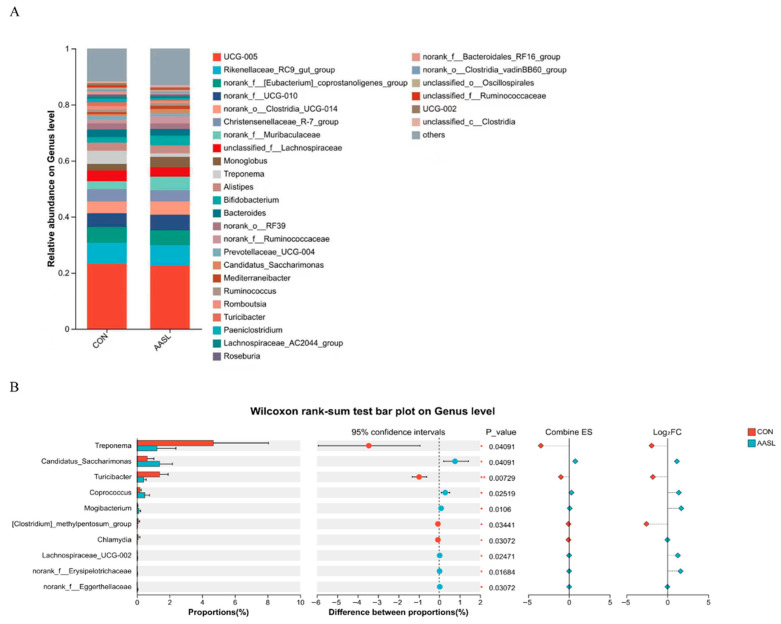
Effects of dietary supplementation with AASL on the genus-level microbial community structure in calves: (**A**) Genus-level microbial community composition. Different colored blocks indicate different taxa, while the gray “others” represents the remaining low-abundance taxa. (**B**) Differential genera of the microbial community at the genus level. Dots indicate the estimated differences, horizontal lines indicate the 95% confidence intervals, diamonds indicate the effect size and Log_2_FC, and the vertical dashed line indicates the zero reference line. The *p*_value column shows the significance test results, and * indicates a significant difference (*p* < 0.05).

Further analysis of differential taxa at the genus level showed that the gut microbiota of both groups was mainly composed of UCG-005, norank_f_[Eubacterium]_coprostanoligenes_group, norank_f_UCG-010, and Rikenellaceae_RC9_gut_group, which were identified as the dominant genera (Figure 9A). Compared with the CON group, the relative abundances of *Treponema*, *Turicibacter*, *Chlamydia*, and [Clostridium]_methylpentosum_group were significantly reduced in the AASL group, whereas *Candidatus _Saccharimonas*, *Coprococcus*, *Mogibacterium*, Lachnospiraceae_UCG-002, norank_f_Erysipelotrichaceae, and norank_f_Eggerthellaceae were significantly increased (Figure 9B).

#### 3.6.5. LESe Analysis of Differential Taxa

**Figure 10:**
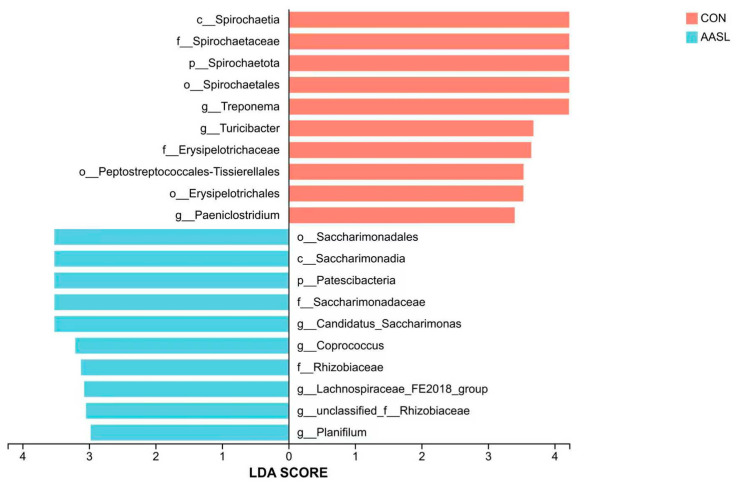
OTU-based LEfSe analysis LDA histogram. LDA, linear discriminant analysis; Red indicates taxa enriched in the CON group, and blue indicates taxa enriched in the AASL group; the x-axis represents the LDA score.

LEfSe analysis from the phylum to genus levels showed distinct taxonomic biomarkers between the two groups (Figure 10). In the CON group, the enriched taxa were c_Spirochaetia, f_Spirochaetaceae, p_Spirochaetota, o_Spirochaetales and g_*Treponema*. In contrast, the AASL group was characterized by the enrichment of o_Saccharimonadales, c_Saccharimonadia, p_Patescibacteria, f_Saccharimonadaceae and g_*Candidatus _Saccharimonas*. These results further indicate that AASL supplementation reshaped the gut microbial composition of calves and promoted the enrichment of specific bacterial taxa distinct from those in the CON group.

#### 3.6.6. Functional Prediction Analysis

**Figure 11:**
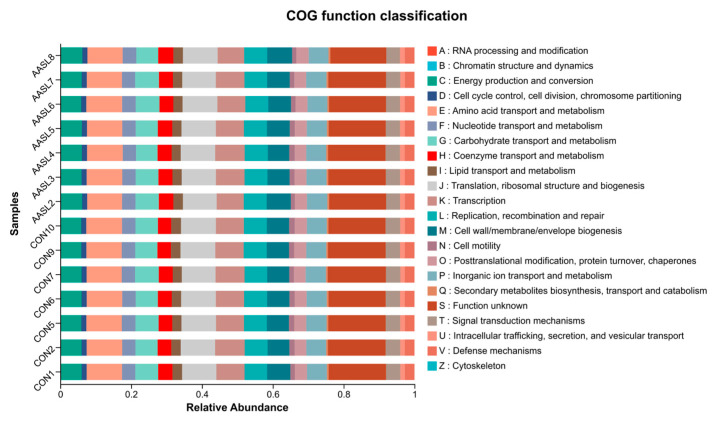
Effects of dietary supplementation with AASL on COG functional prediction analysis in calves. COG, Clusters of Orthologous Groups.The x-axis represents relative abundance, the y-axis represents different samples, and different colors indicate different COG functional categories.

COG-based functional prediction analysis showed that AASL intervention did not markedly alter the core functional profile of the calf gut microbiota (Figure 11). The predominant predicted functions in both groups were mainly related to amino acid transport and metabolism, carbohydrate transport and metabolism, replication, recombination and repair, translation, ribosomal structure and biogenesis, and energy production and conversion, which represent the basic functional potential of the calf intestinal microbiota. The overall functional profiles of the CON and AASL groups were highly similar, and neither the ranking nor the relative abundance of the major functional categories was fundamentally changed. This result was highly consistent with the findings from the alpha and beta diversity analyses, suggesting that although AASL supplementation affected the abundance of specific taxa, it did not substantially disrupt the overall functional stability of the gut microbial community.

### 3.7. Effects of AASL Supplementation on SCFAs Concentrations

**Figure 12:**
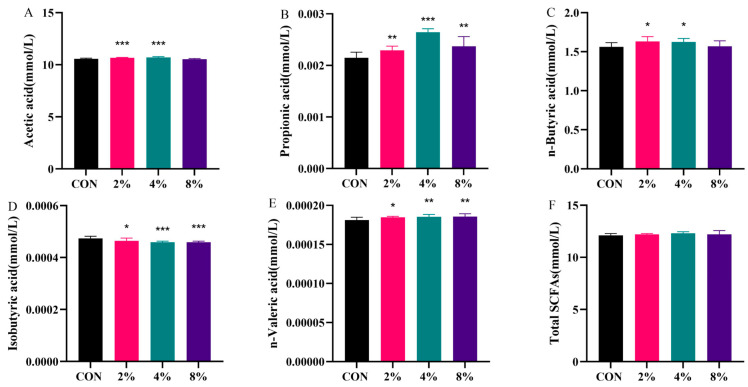
Effects of dietary AASL supplementation on fecal SCFA concentrations in calves: (**A**) Acetic acid; (**B**) Propionic acid; (**C**) n-Butyric acid; (**D**) Isobutyric acid; (**E**) n-Valeric acid; (**F**) Total SCFAs. * Indicates significance at *p* < 0.05, ** indicates significance at *p* < 0.01, and *** indicates significance at *p* < 0.001.

The determination of SCFA contents showed that, compared with the CON group, supplementation with 2% and 4% AASL significantly increased the fecal concentrations of acetic acid and n-butyric acid (*p* < 0.05 or *p* < 0.001). All AASL supplementation levels significantly increased the concentrations of propionic acid, isobutyric acid, and n-valeric acid (*p* < 0.05, or *p* < 0.01, or *p* < 0.001), with the 4% AASL group showing the most pronounced overall effect (Figure 12). The detailed numerical values corresponding to Figure 12 are provided in Supplementary Table S4.

### 3.8. Effects of AASL Supplementation on Untargeted Serum Metabolomics in Calves

#### 3.8.1. Principal Component Analysis

**Figure 13:**
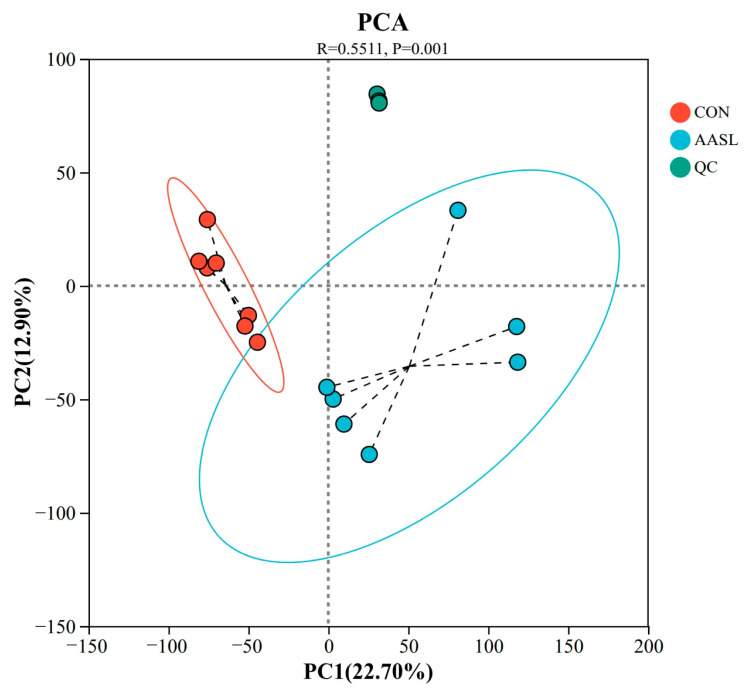
PCA of serum untargeted metabolomics data. PCA, Principal component analysis; Red, blue, and green dots represent the CON, AASL and QC groups, respectively. The red and blue ellipses indicate the distribution ranges of the CON and AASL groups. Black dotted lines indicate sample-to-centroid connections, and gray dashed lines indicate coordinate reference lines. PC1 and PC2 represent the first two principal components with their explained variance, and the R and P values indicate the between-group statistical results.

To evaluate the overall metabolic differences between the CON and AASL groups, as well as the degree of variation within each group, PCA, an unsupervised multivariate statistical method, was performed. The PCA results showed tight clustering of the QC samples, indicating excellent analytical stability and reliable QC in this experiment. Meanwhile, the CON and AASL groups exhibited a clear separation trend, suggesting that AASL intervention may affect the distribution of metabolic profiles (Figure 13).

#### 3.8.2. Partial Least Squares Discriminant Analysis and Permutation Test

**Figure 14:**
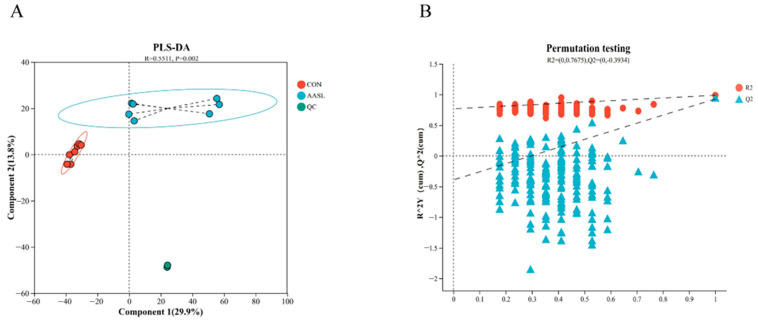
PLS-DA and permutation test model of untargeted serum metabolomics: (**A**) PLS-DA; PLS-DA, Partial least squares discriminant analysis. Red, blue, and green dots represent the CON, AASL and QC groups, respectively; ellipses of different colors indicate the distribution ranges of each group, and black dashed lines represent the connections between samples and the group centroid. The x-axis and y-axis represent Component 1 and Component 2, respectively, together with their explained variance. (**B**) permutation test model. Red circles represent R^2^, blue triangles represent Q^2^, and dashed lines indicate the regression fitting lines; a Q^2^ intercept lower than 0 indicates no obvious overfitting of the model.

To further improve group discrimination and predict sample classification, a PLS-DA model was established for the CON and AASL groups. The PLS-DA results showed a complete separation of metabolite distributions between the two groups in the combined positive and negative ion modes, indicating that AASL treatment markedly altered the serum metabolic profile (Figure 14A). Further validation of the PLS-DA model showed that the R^2^ values were all higher than the corresponding Q^2^ values in the combined ion mode, and the intercept of the Q^2^ regression line with the *Y*-axis was −0.3934, indicating that the model had good fitness and strong predictive ability and was therefore suitable for subsequent data analysis (Figure 14B).

#### 3.8.3. Differential Metabolites

**Figure 15:**
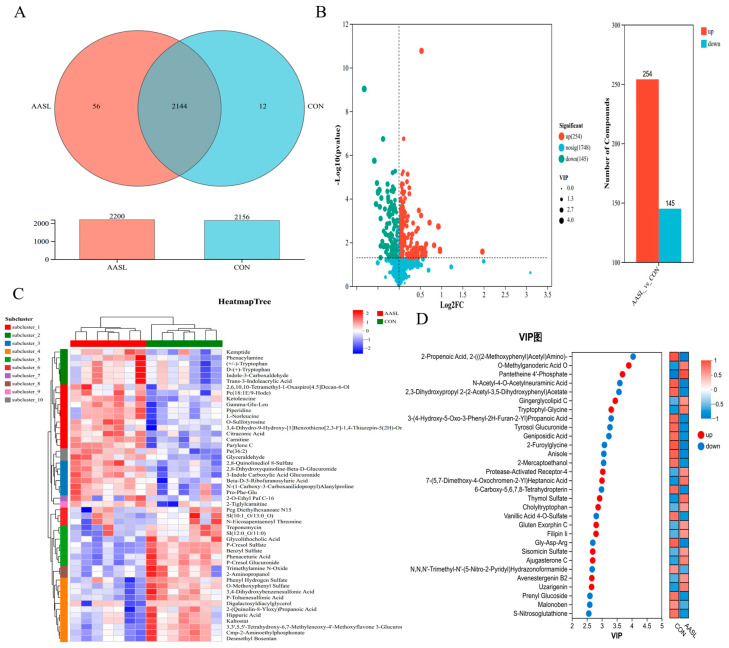
Differential metabolite analysis of untargeted serum metabolomics in the CON and AASL groups: (**A**) Venn diagram showing the numbers of shared and unique metabolites between the CON and AASL groups; The bar plot below the Venn diagram shows the total number of detected metabolites in each group. of metabolites in the CON and AASL groups. (**B**) volcano plot of differential metabolites. Each dot represents one metabolite; red indicates up-regulated metabolites, teal indicates down-regulated metabolites, light blue indicates non-significant metabolites, and dot size represents the VIP value. (**C**) hierarchical clustering analys. Colors ranging from blue to red indicate relative abundance from low to high; the top color strip indicates sample grouping, and the left color strip indicates different metabolite subclusters. (**D**) VIP bar plot of differential metabolites. VIP, variable importance in projection; Red dots indicate up-regulated metabolites, blue dots indicate down-regulated metabolites, and the small heatmap on the right shows the relative abundance changes of each metabolite between the two groups.

To furthercharacterize metabolite composition in the CON and AASL groups, a Venn diagram analysis was performed based on the combined positive and negative ion modes. The results showed that 2156 metabolites were detected in the CON group and 2200 metabolites were detected in the AASL group. Among these, CON specific metabolites accounted for 25.45%, whereas AASL specific metabolites accounted for 0.56% (Figure 15A).

Further differential metabolite analysis showed that, in the comparison between the AASL and CON groups, 1748 metabolites were unchanged, whereas 254 metabolites were upregulated and 145 metabolites were downregulated (Figure 15B). Hierarchical clustering analysis further revealed that these differential metabolites were grouped into 10 distinct clusters under the combined ion mode. Compared with the CON group, clusters 1, 2, 3, 7 and 10 were upregulated in the AASL group, whereas clusters 4, 5, 6, 8 and 9 were downregulated (Figure 15C).

Based on the VIP scores derived from the PLS-DA model under the combined positive and negative ion mode, 30 differential metabolites with VIP > 2 were identified. Among them, representative upregulated metabolites included Pantetheine 4′-phosphate, which is related to coenzyme metabolism, Gingerglycolipid C, which is associated with lipid-related metabolism, and Tryptophyl-Glycine, which is involved in amino acid metabolism. Representative downregulated metabolites included N-Acetyl-4-O-Acetylneuraminic Acid, which is related to glycoconjugate modification, and 3-(4-Hydroxy-5-Oxo-3-Phenyl-2H-Furan-2-Yl) Propanoic Acid (Figure 15D) (Table 5).

#### 3.8.4. Metabolic Pathway Analysis

**Figure 16:**
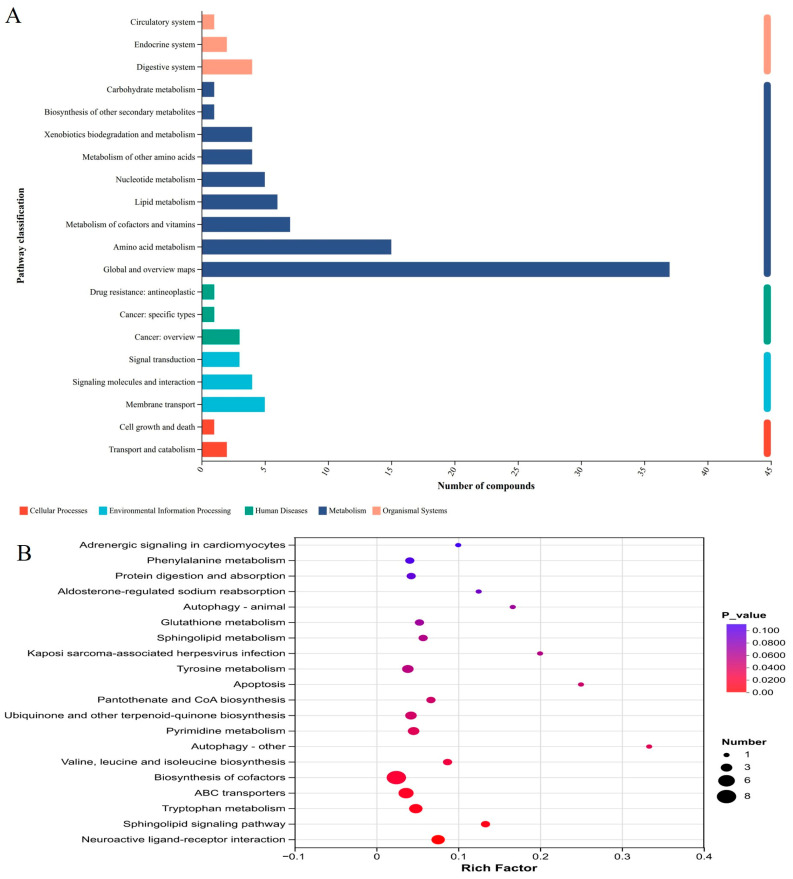
KEGG pathway analysis of untargeted serum metabolomics in the CON and AASL groups: (**A**) KEGG functional classification of metabolites in the CON and AASL groups. The x-axis represents the number of compounds, and the y-axis represents the pathway categories; different colors indicate different KEGG level-1 classifications. groups. (**B**) KEGG enrichment analysis of differential metabolites between the CON and AASL groups.The x-axis represents the rich factor, and the y-axis represents the enriched pathway names; bubble size indicates the number of enriched differential metabolites, and bubble color represents the *p* value, with red indicating higher significance.

To further elucidate the biological pathways associated with the differential metabolites, KEGG enrichment analysis was performed. The KEGG functional classification results showed that the differential metabolites were mainly involved in five major functional categories, including Cellular Processes, Environmental Information Processing, Human Diseases, Metabolism, and Organismal Systems (Figure 16A). Pathway enrichment analysis further revealed that these differential metabolites were significantly enriched in several key metabolic pathways, including neuroactive ligand–receptor interaction, sphingolipid signaling pathway, tryptophan metabolism, ABC transporters and biosynthesis of cofactors (Figure 16B).

### 3.9. Correlation Analysis 

#### 3.9.1. Correlation Analysis Between Gut Microbiota and SCFAs

**Figure 17:**
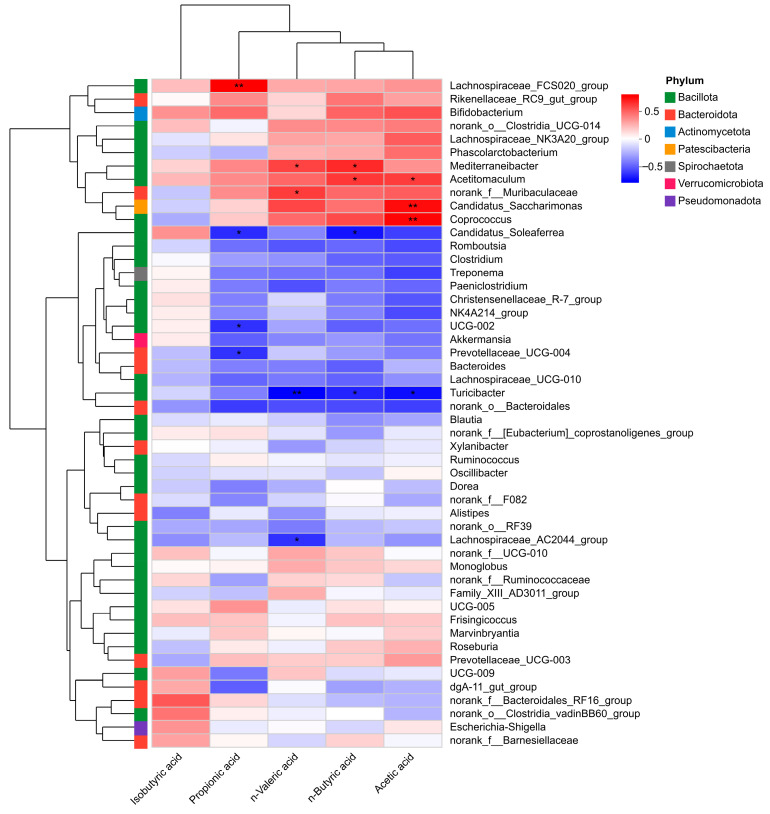
Correlation analysis between calves gut microbiota and SCFAs. Red indicates positive correlation, blue indicates negative correlation, and color intensity reflects correlation strength. The dendrograms on the left and top indicate clustering relationships of taxa and SCFAs, respectively, and the colored strip on the left indicates the phylum-level classification of each taxon. * *p* < 0.05, ** *p* < 0.01.

Spearman’s correlation analysis was performed to evaluate the associations between genus-level gut microbiota and five SCFAs (Figure 17). Significant correlations were screened based on *p* values (*p* < 0.05, significant; *p* < 0.01, highly significant). The results showed that propionic acid was positively correlated with *Lachnospiraceae_FCS020_group* (*p* = 0.00348), and negatively correlated with *Prevotellace*ae_UCG-004 (*p* = 0.04282), Prevotellavceae_UCG-*002* (*p* = 0.04085) and *Candidatus_Soleaferrea* (*p* = 0.03533). n-Valeric acid was positively correlated with *Mediterraneibacter* (*p* = 0.04916) and *norank_f_Muribaculaceae* (*p* = 0.04085), and negatively correlated with *Lachnospiraceae_AC*2044_group (*p* = 0.04085) and *Turicibacter* (*p* = 0.00563). n-Butyric acid was positively correlated with *Mediterraneibacter* (*p* = 0.02461) and *Acetitomaculum* (*p* = 0.0371), and negatively correlated with *Candidatus_Soleaferrea* (*p* = 0.01755) and *Turicibacter* (*p* = 0.02885). Acetic acid was positively correlated with *Acetitomaculum* (*p* = 0.04085), *Coprococcus* (*p* = 0.00721) and *Candidatus_Saccharimonas* (*p* = 0.00932), and negatively correlated with *Turicibacter* (*p* = 0.01466).

#### 3.9.2. Correlation Analysis Between Gut Microbiota and Metabolite

**Figure 18:**
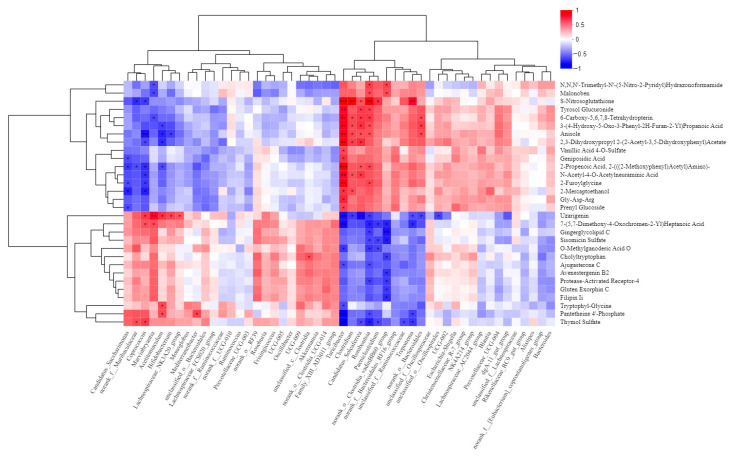
Correlation analysis between calf gut microbiota and metabolites. The right side of the figure shows metabolite names, and the bottom shows gut microbiota. Red indicates a positive correlation, blue indicates a negative correlation, and color intensity reflects the strength of the correlation. The dendrograms on the left and top represent the clustering relationships of metabolites and microbiota, respectively. * *p* < 0.05, ** *p* < 0.01, *** *p* < 0.001.

Correlation analysis was conducted between the top 30 differential metabolites ranked by VIP values and the gut microbiota at the genus level, and the results are presented in Figure 18. The heatmap revealed two distinct and opposing correlation clusters. The bacterial taxa clustered in the upper part of the heatmap showed strong positive correlations with one group of metabolites but strong negative correlations with another, whereas the taxa in the lower cluster displayed the opposite pattern, indicating a marked antagonistic association structure. *Candidatus_Saccharimonas*, Muribaculaceae, *Coprococcus*, *Marvinbryantia*, Rikenellaceae, *Bifidobacterium*, and Actinobacteriota were significantly positively correlated with malonate, S-nitrosoglutathione, tyrosol glucuronide, and vanillic acid 4-O-sulfate, which are mainly associated with antioxidant, anti-inflammatory, and aromatic metabolism (r ≈ 0.5 to 1.0). In contrast, these taxa were significantly negatively correlated with uzarigenin, gingerolyside C, and sisomicin sulfate, which are primarily related to steroid-like, antibiotic-related, and neuroactive metabolites (r ≈ −1.0 to −0.5). Conversely, Escherichia-Shigella, Lachnospiraceae_R7_group, NK4A214_group, *Blautia*, and Proteobacteria exhibited the opposite correlation pattern, showing significant positive correlations with steroid-like, antibiotic-related, and neuroactive metabolites, while being significantly negatively correlated with antioxidant, anti-inflammatory, and aromatic metabolites.

## 4. Discussion

Long regarded as agricultural waste, the stems and leaves of *Astragalus membranaceus* and *Angelica sinensis* are now attracting increasing attention because they still contain abundant saponins, polysaccharides, flavonoids, nutrients and trace elements, with some constituents in *Astragalus membranaceus* stems and leaves even exceeding those in the medicinal root [14]. Similarly, the *Angelica sinensis* stems and leaves contain bioactive compounds comparable to those of the root [15]. Owing to their abundant availability, low cost, lack of drug residues, and dual medicinal–nutritional value, these plant materials have considerable potential as functional feed ingredients. Under intensive production systems, calves are often exposed to multiple stressors, including immature immunity, weaning stress, and pathogen challenge, which can induce gut dysbiosis, reduce antioxidant enzyme activity, and trigger excessive inflammatory responses [16,17,18]. Because the intestine is a major immune organ and diet is one of the key determinants of gut microbial composition, early-life nutritional interventions may have important implications for calf health, later productivity, and economic efficiency [19,20,21]. Therefore, this study evaluated the potential of AASL as a medicinal feed supplement by examining its effects on serum antioxidant, inflammatory, and immune indices, as well as gut microbiota and serum metabolites in Simmental bull calves.

The major bioactive constituents and nutritional components of AASL were quantitatively analyzed in the present study. The results showed that the contents of total sugars, total flavonoids, and total saponins were 2.24 ± 0.09%, 0.12 ± 0.01%, and 1.22 ± 0.40%, respectively, on a powder basis. These findings provide a material basis for the antioxidant, anti-inflammatory, and immunomodulatory effects observed subsequently in this study. However, the active constituents in AMSL and ASSL were not determined separately. Previous studies have shown that AMSL are rich in bioactive compounds, particularly polysaccharides and flavonoids, with total polysaccharide contents generally ranging from 335 to 552.5 mg/g [22] and total flavonoid contents ranging from 11.449 to 15.008 mg/g [23]. ASSL have likewise been reported to contain abundant flavonoids, with levels ranging from 19.4531 to 91.5685 mg/g [24]. During diet formulation, AASL was incorporated at the predetermined proportion to replace part of the basal fiber sources, aiming to keep total energy and nitrogen content of the rations as consistent as possible across groups. Nutritional analysis showed that AASL was rich in crude protein, crude fat, and trace elements, suggesting that it may help optimize dietary nutrient balance and improve feed utilization. These nutrients and bioactive components may also promote animal growth and health, particularly because trace elements are closely associated with immune function and antioxidant capacity [25,26]. The ADG of calves in the 4% AASL supplementation group was significantly increased, whereas no significant differences were observed in the other dosage groups. This suggests that AASL may promote the growth of post-weaning calves when provided at an appropriate supplementation level. The lack of significant effects in the 2% and 8% groups further indicates that a lower dose may be insufficient to fully exert its biological activity, whereas a higher dose may fail to produce additional growth-promoting effects due to factors such as palatability, metabolic burden, or saturation of the active components.

In the present study, supplementation with 4% AASL tended to increase serum IgA, IgM, and IgG levels in calves, suggesting that AASL may exert a positive effect on humoral immunity. This finding is generally consistent with previous reports showing that phytogenic feed additives can improve immune related parameters in animals [27]. At the same time, AASL reduced the serum levels of TNF-α, IL-6, and IL-1β, with the 4% supplementation group showing more pronounced changes in these inflammatory markers, suggesting that AASL may help alleviate inflammatory responses in calves. Following AASL supplementation, IL-4 levels showed an increasing trend, although the change did not reach statistical significance, suggesting that AASL may have a certain tendency to modulate anti-inflammatory cytokines, but that this effect was not stable under the conditions of the present study. In light of the significant decreases observed in TNF-α, IL-6, and IL-1β, the regulatory effect of AASL on inflammatory status may be reflected more in the suppression of pro-inflammatory responses, whereas its promoting effect on anti-inflammatory cytokines such as IL-4 appears to be relatively limited. In addition, AASL significantly increased serum T-AOC, SOD, and GSH activity, suggesting that it may partly regulate immune-related status by enhancing antioxidant defense capacity. This result is also broadly consistent with previous studies showing that plant-derived functional compounds can modulate inflammatory cytokine expression to some extent, and that mixed Chinese herbal extracts may improve immune- and inflammation-related indices in calves [28]. Taken together, these findings suggest that AASL may contribute to improved immune-related status in calves, at least in part, through enhancing antioxidant capacity and modulating inflammatory responses.

The gut microbiota serves as an important hub linking nutrient metabolism and immune regulation. Through nutrient degradation, SCFA production, and maintenance of the mucosal barrier, it forms a complex interaction network with host antioxidant capacity and inflammatory homeostasis [29]. Meanwhile, the serum metabolome can act as a sensitive molecular readout of physiological and biochemical status under nutritional intervention [30], and integrated microbiome–metabolome analysis has become an important approach for identifying biomarkers and elucidating potential biological mechanisms [31]. Previous studies have shown that alterations in the gut microbiota are associated with the remodeling of both local and systemic immune responses, whereas microbial homeostasis contributes to the maintenance of host immune balance [32,33]. In addition, *Candidatus_Saccharimonas* has been reported to be associated with intestinal function maintenance and immune recovery [34], whereas *Treponema* has been linked to pathogenicity and disease-related processes [35]. In the present study, AASL supplementation was associated with marked changes in the abundance of several bacterial genera, suggesting a potential role in reshaping gut microbial composition. As important microbial metabolites, SCFAs have been reported to be closely associated with barrier integrity, glucose and lipid metabolism, as well as the regulation of immune and inflammatory responses [36]. They may also influence the expression of pro-inflammatory cytokines such as TNF-α, IL-12, and IL-6 through macrophage related regulation [37]. In the present study, SCFA levels increased following AASL supplementation, and this finding was consistent with the observed shifts in gut microbial composition and the improvement in inflammation-related indices. However, the specific causal relationships among these phenomena still require further investigation.

Correlation analysis further revealed a clear association between genus-level microbiota and SCFAs. These findings suggest that AASL may improve intestinal fermentation capacity by enriching SCFA associated taxa and suppressing bacteria unfavorable to SCFA accumulation. SCFAs, especially acetate, propionate, and butyrate, are not only major microbial fermentation end-products but also key signaling molecules involved in intestinal barrier function, mucosal immunity, and metabolic homeostasis in young ruminants [13]. Among these, *Coprococcus* can downregulate the expression of pro-inflammatory cytokines TNF-α, IL-1β and IL-6, significantly upregulate the secretion of anti-inflammatory cytokines IL-4, IL-5 and IL-10, promote the maturation of intestinal goblet cells and mucin expression, restore the levels of tight junction proteins such as claudin-1, occludin, and ZO-1, and maintain intestinal barrier integrity. Furthermore, as a SCFAs-producing bacterium, the main metabolite of *Coprococcus* is acetic acid [38].

Metabolomics is a powerful systems biology tool for characterizing small-molecule metabolic alterations and is widely used to clarify drug efficacy and mechanisms [39]. In this study, AASL markedly altered the metabolic profile of calves, including increased levels of energy-related intermediates such as Pantetheine 4′-phosphate and neuroactive metabolites such as Tryptophyl-Glycine, suggesting possible effects on energy metabolism, microbiota–host co-metabolism and the neuroimmune axis [40]. KEGG enrichment showed that differential metabolites were mainly involved in lipid and carbohydrate metabolism, which are central to energy homeostasis and nutrient utilization. Notably, tryptophan metabolism, the precursor pathway for serotonin biosynthesis, is closely linked to intestinal motility, secretion and neuroimmune regulation [41], and microbiota-derived tryptophan metabolites may also influence neuroinflammation and gut–brain axis signaling [42]. Sphingolipids, another enriched category, are bioactive membrane lipids involved in apoptosis, migration, aging, and inflammation [43]. Together, these findings indicate that AASL exerts multilayered regulatory effects on host metabolism rather than acting on a single pathway [44]. Pathway analysis further showed enrichment in neuroactive ligand–receptor interaction, sphingolipid signaling, tryptophan metabolism, ABC transporters, and cofactor biosynthesis, all of which are recognized as important nodes in gut microbiota–host interactions [45]. Energy metabolism pathways, especially those linked to the tricarboxylic acid cycle, are also closely associated with immune cell function and inflammatory regulation [46]. These results suggest that AASL may regulate host metabolic networks through multiple targets, particularly by improving nutrient utilization, maintaining energy homeostasis, and alleviating inflammatory imbalance.

Correlation analysis between the top 30 differential metabolites ranked by VIP values and the gut microbiota at the genus level showed that microbiota–metabolite interactions were not randomly dispersed, but instead formed two oppositely directed correlation modules. One group of bacteria varied in the same direction as antioxidant, anti-inflammatory, and aromatic metabolites, while varying in the opposite direction to steroid-like, antibiotic-like, and some neuroactive metabolites. Another group of bacteria exhibited the exact opposite pattern. Among these taxa, *Bifidobacterium* is widely recognized as an important probiotic for maintaining intestinal homeostasis, exerting protective effects through the production of acetate and lactate, promotion of mucus layer stability, and regulation of host immune responses [47]. *Coprococcus* is a typical butyrate associated genus, and its reduction is often associated with inflammatory conditions or impaired health status [48]. In recent years, *Muribaculaceae* has also been considered closely related to polysaccharide utilization, SCFA production, maintenance of intestinal barrier integrity, and immune regulation [49]. Therefore, the clustering of these taxa within the same correlation module as antioxidant and anti-inflammatory metabolites suggests that they may jointly contribute to maintaining a lower inflammatory state and a more stable intestinal microenvironment. Tyrosol and its conjugated metabolites generally possess antioxidant and anti-inflammatory activities, while vanillic acid and its derivatives have also been shown to have the potential to scavenge reactive oxygen species and suppress inflammatory responses [50]. Thus, the co-occurrence of these metabolic signals with beneficial bacteria within the same module indicates that this module may represent a metabolic network oriented toward antioxidant defense and inflammatory buffering. In contrast, the expansion of Proteobacteria has long been regarded as an important hallmark of gut microbiota dysbiosis, and increased abundance of Escherichia-Shigella is commonly associated with impaired intestinal barrier function, endotoxemia, and an aggravated inflammatory environment. Therefore, the negative correlation between this group of bacteria and protective metabolites may reflect a reduction in the host’s antioxidant and antiinflammatory metabolic reserves under an unfavorable microbial ecological state.

## 5. Conclusions

The AASL shows promising potential as a functional feed additive for weaned Simmental bull calves. Dietary supplementation with AASL can modulate immune-related parameters, antioxidant status, inflammatory cytokine levels, gut microbiota composition, fecal SCFA concentrations, and serum metabolic profiles. Under the conditions of the present study, 4% AASL may be considered the recommended supplementation level.

From a practical production perspective, AASL may serve as a promising functional feed additive candidate during the post-weaning period, particularly for calves experiencing greater weaning stress or requiring improved health management. It may be especially valuable in antibiotic-reduction or antibiotic-free production systems for improving calf health and maintaining stable production performance. Nevertheless, further refined dose–response studies and on-farm validation are still needed to define the optimal supplementation range more precisely and to support its wider practical application.

## Figures and Tables

**Table 1:** Nutritional composition of *Astragalus membranaceus* stem and leaf–*Angelica sinensis* stem and leaf (AASL) and the basal diet (%Dry Matter).

Item	AASL	Concentrate Feed	Corn Silage	Wheat Straw
Dry Matter (DM)	91.32	88.95	90.12	91.15
Crude Protein (CP)	14.91	11.56	8.31	2.42
Ether Extract (EE)	2.75	3.21	3.01	1.91
Crude Ash (Ash)	4.83	5.8	5.71	9.15
Calcium (Ca)	1.59	0.46	0.23	0.2
Sodium (Na)	0.30	0.644	0.11	/
Magnesium (Mg)	0.41	0.26	0.29	/
phosphorus (P)	0.15	0.56	0.27	0.16
Acid Detergent Fiber (ADF)	34.09	4.11	18.58	50.75
Neutral Detergent Fiber (NDF)	45.54	9.44	33.44	72.58

**Table 2:** Ingredient composition of the experimental diets (% of diet DM).

Ingredient	CON	2%AASL	4%AASL	8%AASL
Concentrate feed	69.4	69.4	69.4	69.4
Corn silage	18.75	17.52	16.3	13.85
Wheat straw	11.85	11.08	10.3	8.75
AASL	0	2	4	8
Total	100	100	100	100

**Table 3:** Contents of total sugars, total flavonoids, and total saponins in AASL.

Active Fractions	Content (%)
Total Sugars	2.24 ± 0.09
Total Flavonoids	0.12 ± 0.01
Total Saponins	1.22 ± 0.40

**Table 4:** Effects of dietary supplementation with AASL on growth performance of calves.

Item	CON Group	2%AASL	4%AASL	8%AASL
Initial body weight (kg)	141.79 ± 14.57	143.43 ± 13.09	138.93 ± 8.56	131.86 ± 7.62
Final body weight (kg)	155.29 ± 15.14	154.93 ± 14.07	154.80 ± 9.82	146.43 ± 8.63
Average daily gain (kg)	0.90 ± 0.08	0.87 ± 0.19	1.05 ± 0.16 *	0.97 ± 0.14

Compared with the control group, * indicates a significant difference (*p* < 0.05).

**Table 5:** List of Key Differential Metabolites Identified by PLS-DA Between AASL and CON Groups.

Metabolite	Metab ID	VIP	*p*-Value
2-Propenoic Acid, 2-(((2-Methoxyphenyl) Acetyl) Amino)-	metab_20146	4.0442	9.43 × 10^−10^
O-Methylganoderic Acid O	metab_8354	3.898	0.001855
Pantetheine 4′-Phosphate	metab_14111	3.689	1.70 × 10^−11^
N-Acetyl-4-O-Acetylneuraminic Acid	metab_2694	3.6007	1.82 × 10^−6^
2,3-Dihydroxypropyl 2-(2-Acetyl-3,5-Dihydroxyphenyl) Acetate	metab_20452	3.5708	3.96 × 10^−5^
Gingerglycolipid C	metab_2498	3.4485	0.02112
Tryptophyl-Glycine	metab_13701	3.3133	0.001229
3-(4-Hydroxy-5-Oxo-3-Phenyl-2H-Furan-2-Yl) Propanoic Acid	metab_9317	3.3098	4.73 × 10^−5^
Tyrosol Glucuronide	metab_19994	3.2701	5.34 × 10^−5^
Geniposidic Acid	metab_20587	3.2357	0.0001752
2-Furoylglycine	metab_12836	3.0867	1.83 × 10^−7^
Anisole	metab_9282	3.0658	1.89 × 10^−5^
2-Mercaptoethanol	metab_829	3.0585	0.0002338
Protease-Activated Receptor-4	metab_2530	3.031	0.0118
7-(5,7-Dimethoxy-4-Oxochromen-2-Yl) Heptanoic Acid	metab_9625	3.0044	0.0005812
6-Carboxy-5,6,7,8-Tetrahydropterin	metab_9313	2.9883	9.21 × 10^−5^
Thymol Sulfate	metab_15271	2.9255	0.0003412
Cholyltryptophan	metab_2400	2.8744	0.02306
Vanillic Acid 4-O-Sulfate	metab_20481	2.8133	0.0008033
Gluten Exorphin C	metab_1698	2.8098	0.02083

## Data Availability

The original contributions presented in this study are included in the article/Supplementary Material. Further inquiries can be directed to the corresponding authors.

## Supplementary Materials

The following supporting information can be downloaded at: https://www.mdpi.com/article/10.3390/vetsci13050414/s1, Table S1: Hematological parameters in each experimental group; Table S2: Immunoglobulin and inflammatory cytokine levels in each experimental group; Table S3: Antioxidant indices in each experimental group; Table S4: SCFA levels in each experimental group.

## Abbreviations

The following abbreviations are used in this manuscript:

AASL	*Angelica membranaceus* stem and leaf–*Astragalus sinensis* stem and leaf mixture
ADG	Average daily gain
IgA	Immunoglobulin A
PLS-DA	Partial least squares discriminant analysis
AMSL	*Astragalus membranaceus* stem and leaf
T-AOC	Total antioxidant capacity
LDH	Lactate dehydrogenase
LYM	Lymphocyte count
ASSL	*Angelica sinensis* stem and leaf
MDA	Malondialdehyde
Gran	Granulocyte count
OTUs	Operational taxonomic units
IgG	Immunoglobulin G
SCFAs	Short-chain fatty acids
SOD	Superoxide dismutase
PCA	Principal component analysis
IgM	Immunoglobulin M
TNF-α	Tumor necrosis factor-alpha
VIP	Variable importance in projection
WBC	White blood cell count
