# Supplementary material for: Effects of an Astragalus membranaceus Stem and Leaf-Angelica sinensis Stem and Leaf Mixture on Serum Parameters, Gut Microbiota, and Metabolomic Profiles in Simmental Weaned Bull Calves
Source: Vet Sci. 2026 Apr 23;13(5):414. doi: 10.3390/vetsci13050414 (PMC13211713; doi:10.3390/vetsci13050414)
Supplement: Supplementary file 1 [file vetsci-13-00414-s001.zip › vetsci-4236056-supplementary.pdf]

Supplementary Materials for [Effects of an *Astragalus membranaceus* Stem and Leaf *Angelica sinensis* Stem and Leaf Mixture on Serum Parameters, Gut Microbiota, and Metabolomic Profiles in Simmental Weaned Bull Calves]

Table S1. Hematological parameters in each experimental group.

Indicator	CON group	2% AASL	4% AASL	8% AASL
WBC (10^9/L)	6.693 ± 0.6987	6.254 ± 0.6947	6.028 ± 0.5528*	6.430 ± 0.8534
GRAN (10^9/L)	2.397 ± 0.2599	2.242 ± 0.2902	2.179 ± 0.2351*	1.911 ± 0.2322***
LYM (10^9/L)	3.914 ± 0.4782	3.187 ± 0.5175***	3.054 ± 0.4060***	3.302 ± 0.5404**
MON (10^9/L)	0.5015 ± 0.0482	0.4100 ± 0.0525***	0.3740 ± 0.0365***	0.4180 ± 0.0565***

Note: Data are presented as Mean ± SD, with four significant figures. Compared with the CON group: * P<0.05, ** P <0.01, *** P <0.001.

Table S2. Immunoglobulin and inflammatory cytokine levels in each experimental group.

Indicator	CON group	2% AASL	4% AASL	8% AASL
IgA(μg/mL)	321.9 ± 20.90	348.1 ± 25.84	362.5 ± 22.82**	349.1 ± 16.12
IgG(mg/mL)	3.109 ± 0.2468	4.962 ± 1.298*	5.830 ± 0.5524***	5.497 ± 1.075**
IgM(μg/mL)	52.15 ± 6.861	71.22 ± 9.103**	74.35 ± 17.08**	66.52 ± 7.755
TNF-α(pg/mL)	17.61 ± 3.453	7.990 ± 1.631***	7.882 ± 1.740***	6.881 ± 2.042***
IL-4(pg/mL)	2.771 ± 0.6189	3.069 ± 0.8925	3.176 ± 0.6724	3.449 ± 1.128
IL-1β(pg/mL)	220.5 ± 17.82	181.6 ± 23.90*	176.0 ± 33.28**	191.6 ± 27.18
IL-6(pg/mL)	66.72 ± 8.403	55.55 ± 5.765*	54.46 ± 8.634**	59.36 ± 7.677

See Table S1 for significance notation.

Table S3. Antioxidant indices in each experimental group.

Indicator	CON group	2% AASL	4% AASL	8% AASL
MDA (nmol/mL)	6.761 ± 1.329	5.545 ± 0.7280	4.679 ± 1.074**	6.392 ± 2.255
SOD (U/mL)	20.97 ± 1.271	24.45 ± 0.9254***	23.66 ± 0.9660***	23.63 ± 1.018***
LDH (U/L)	449.5 ± 12.70	434.7 ± 13.47	431.1 ± 18.12*	432.7 ± 10.50
GSH (μmol/L)	23.18 ± 3.974	29.40 ± 2.952**	33.62 ± 6.168***	34.31 ± 3.347***
TAOC (mM)	0.4347 ± 0.0170	0.4769 ± 0.0082***	0.5090 ± 0.0510**	0.5047 ± 0.0175***
CAT (U/mL)	10.01 ± 0.8549	12.47 ± 1.802**	10.65 ± 2.176	11.35 ± 1.217

See Table S1 for significance notation.

Table S4 Short-chain fatty acid (SCFA) levels in each experimental group.

Indicator	CON group	2% AASL	4% AASL	8% AASL
Acetic acid(mmol/L)	10.55 ± 0.0730	10.67 ± 0.0381***	10.69 ± 0.0886***	10.54 ± 0.0609
Propionic acid (μmol/L)	2.146 ± 0.1089	2.293 ± 0.08019**	2.644 ± 0.06753***	2.368 ± 0.1892**
n-Butyric acid (mmol/L)	1.563 ± 0.05302	1.631 ± 0.06078*	1.624 ± 0.04487*	1.570 ± 0.06951
Isobutyric acid (μmol/L)	0.4745 ± 0.0779	0.4641 ± 0.0113*	0.4593 ± 0.0041***	0.4593 ± 0.0041***
n-Valeric acid (μmol/L)	0.1811 ± 0.0036	0.1845 ± 0.0013*	0.1853 ± 0.0031**	0.1855 ± 0.0039**
Total SCFAs (mmol/L)	12.10 ± 0.1846	12.21 ± 0.0714	12.30 ± 0.1658	12.19 ± 0.3798

See Table S1 for significance notation.
